# Supplementary material for: Mycobacterium tuberculosis PE_PGRS19 Induces Pyroptosis through a Non-Classical Caspase-11/GSDMD Pathway in Macrophages
Source: Microorganisms. 2022 Dec 14;10(12):2473. doi: 10.3390/microorganisms10122473 (PMC9785159; doi:10.3390/microorganisms10122473)
Supplement: Supplementary file 1 [file microorganisms-10-02473-s001.zip › microorganisms-2095839-supplementary.pdf]

Supplementary Materials:

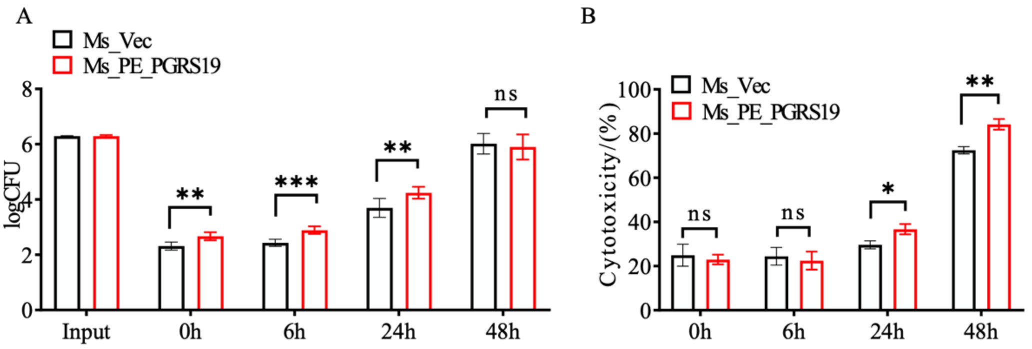

Figure S1. PE_PGRS19 strengthened the intracellular survival of *Mycobacterium smegmatis* and host cell toxicity. (A) The intracellular survival rate after infecting Raw264.7 cells with Ms_PE_PGRS19 and Ms_Vec was estimated by counting CFUs at 0, 6, 24 and 48 h after clearance of extracellular bacteria. (B) Cell toxicity was estimated by measuring LDH secretion in the supernatant of Ms_PE_PGRS19- and Ms_Vec-infected J774A.1 cells at 0, 6, 24 and 48 h after clearance of extracellular bacteria. Multiple t-tests and 2-way ANOVA analyses were performed using the Holm–Šídák method. Data presented as $\bar{x} \pm SD$ of three independent experiments. Ns $p > 0.05$, *$p < 0.05$, **$p < 0.01$, ***$p < 0.001$.

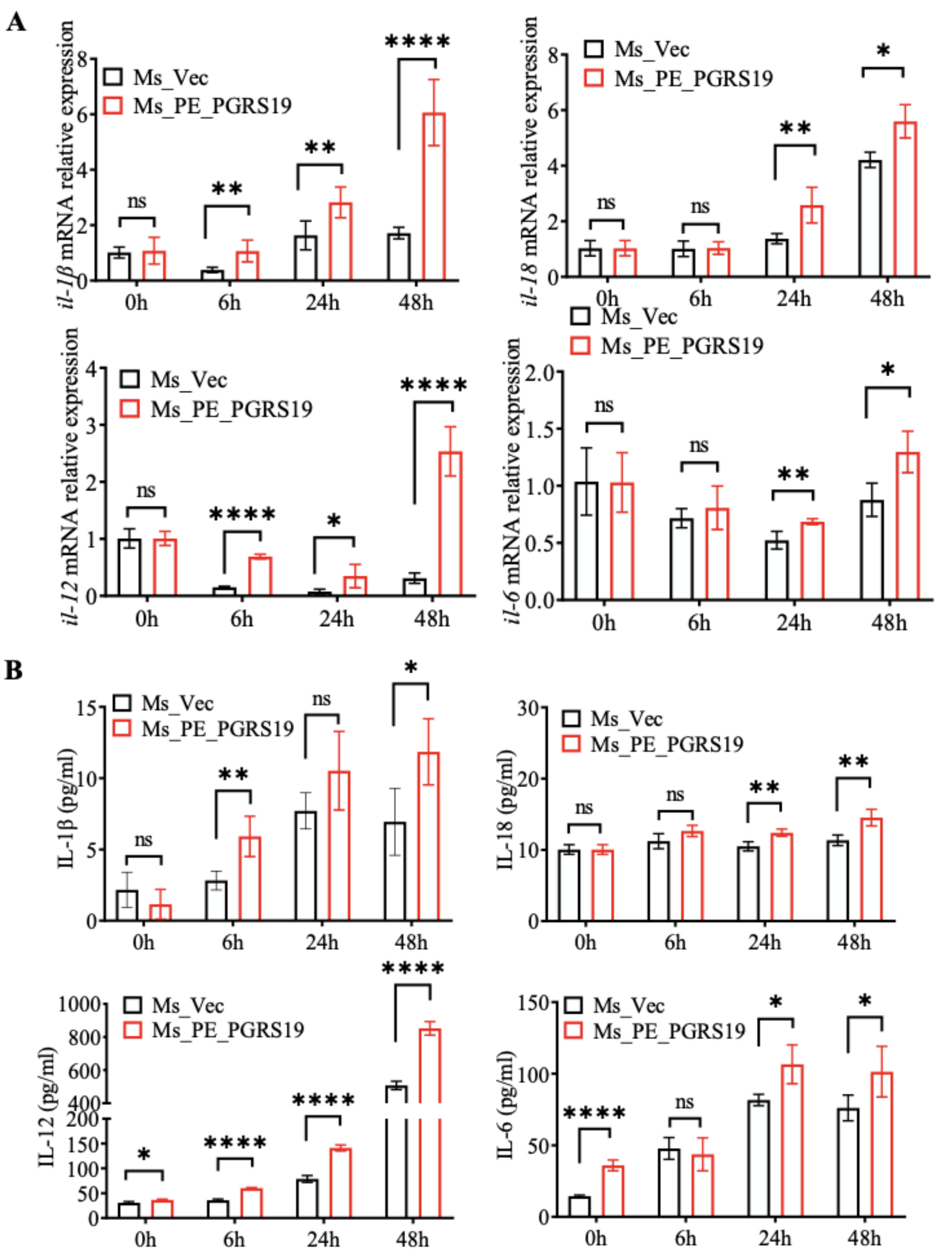

Figure S2. Cell cytokine expression and secretion in Raw264.7 cells infected with the recombinant strains. After Ms_PE_PGRS19 and Ms_Vec infected cells for 4 h, gentamicin was added for 2 h to remove extracellular bacteria, and the time to remove extracellular bacteria was set to 0 h. (A) Relative expression of the *il-1β*, *il-6*, *il-12,* and *il-18* genes in Raw264.7 cells after infection with Ms_PE_PGRS19 and Ms_Vec at 0, 6, 24 and 48 h. (B) Secretion of IL-6, IL-12, IL-1β and IL-18 in Raw264.7 cell culture supernatant was measured by ELISA after infection with Ms_PE_PGRS19 and Ms_Vec at 0, 6, 24 and 48 h. Data are presented as $\bar{x} \pm$ SD of three independent experiments. Ns $p > 0.05$, *$p < 0.05$, **$p < 0.01$, ****$p < 0.0001$.
